# Physical and mental health of older people while cocooning during the COVID-19 pandemic

**DOI:** 10.1093/qjmed/hcab015

**Published:** 2021-01-20

**Authors:** L Bailey, M Ward, A DiCosimo, S Baunta, C Cunningham, R Romero-Ortuno, R A Kenny, R Purcell, R Lannon, K McCarroll, R Nee, D Robinson, A Lavan, R Briggs

**Affiliations:** 1 From the Mercer’s Institute for Successful Ageing, St James’s Hospital, Dublin 1, Ireland; 2 The Irish Longitudinal Study on Ageing, Trinity College, Dublin 1, Ireland; 3 Department of Medical Gerontology, School of Medicine, Trinity College Dublin, Dublin 1, Ireland

## Abstract

**Background:**

Cocooning or shielding, i.e. staying at home and reducing face-to-face interaction with other people, was an important part of the response to the COVID-19 pandemic for older people. However, concerns exist regarding the long-term adverse effects cocooning may have on their physical and mental health.

**Aim:**

To examine health trajectories and healthcare utilization while cocooning in a cohort of community-dwelling people aged ≥70 years.

**Design:**

Survey of 150 patients (55% female, mean age 80 years and mean Clinical Frailty Scale Score 4.8) attending ambulatory medical services in a large urban university hospital.

**Methods:**

The survey covered four broad themes: access to healthcare services, mental health, physical health and attitudes to COVID-19 restrictions. Survey data were presented descriptively.

**Results:**

Almost 40% (59/150) reported that their mental health was ‘worse’ or ‘much worse’ while cocooning, while over 40% (63/150) reported a decline in their physical health. Almost 70% (104/150) reported exercising less frequently or not exercising at all. Over 57% (86/150) of participants reported loneliness with 1 in 8 (19/150) reporting that they were lonely ‘very often’. Half of participants (75/150) reported a decline in their quality of life. Over 60% (91/150) agreed with government advice for those ≥70 years but over 40% (61/150) reported that they disliked the term ‘cocooning’.

**Conclusions:**

Given the likelihood of further restrictions in coming months, clear policies and advice for older people around strategies to maintain social engagement, manage loneliness and continue physical activity and access timely medical care and rehabilitation services should be a priority.

## Introduction

In terms of morbidity and mortality, COVID-19 disproportionately affects frail, older people. Older people with COVID-19 are more likely to develop severe respiratory illness^1^ and delirium.^2^ In Ireland, almost 80% of deaths from COVID-19 have involved people aged ≥75 years^3^ and the crude mortality proportion in people aged 70–79 years with COVID-19 is almost 23%, rising to almost 30% in those aged ≥80 years.^4^

In order to reduce the risk of contracting COVID-19, in late March 2020 all people aged ≥70 years (as well as some younger people with underlying health conditions) in Ireland were advised to stay at home and reduce face-to-face interaction with other people as much as possible.^3^ Older people were advised to stay indoors, have groceries and medicines delivered and avoid contact with friends and family in order to minimize spread within a high-risk group, delay peaks in case numbers and relieve pressure on health services. The term most commonly used to describe this strategy of self-isolation in Ireland was ‘cocooning’,^5^ however alternative terms such as shielding or sheltering have also been used to describe similar strategies involving older populations in other countries.^6^^,^^7^ Cocooning recommendations remain in place at this current time, but there have been some relaxations since they were introduced, involving for example, that shopping is now allotted to designated hours and support bubbles for those living alone are now recommended.

Social isolation, an inevitable consequence of cocooning for many older people, can have a profound impact longitudinally on health in later life. Indeed, socially isolated older people are more likely to report loneliness^8^ and disturbed sleep,^9^ have a higher likelihood of developing depression and psychological distress,^10^ engage in less physical activity and more sedentary time,^11^ demonstrate unhealthy behaviors in general^12^ and have premature mortality.^13^

Other potential consequences of cocooning, including increased sedentary behavior and lack of physical exercise,^14^ reduction in leisure activities^15^ and restricted access to important services can also adversely affect health in later life.^16^

Therefore, while cocooning forms an important part of the public health response to the COVID-19 pandemic, with an overall aim to prevent transmission to vulnerable older people, concerns exist regarding the long-term adverse effects it may have on their physical and mental health. The aim of this study was to examine trajectories of physical and mental health, access to important services and activities and healthcare utilization while cocooning in a cohort of community-dwelling people aged ≥70 years attending ambulatory services in a large university teaching hospital.

## Materials and methods

### Study design and setting

A questionnaire was administered to community-dwelling older people attending older person-specific ambulatory care services in a large teaching hospital.

St James’s Hospital is a large urban teaching hospital with a well-developed ambulatory care service for older people. Participants were recruited from general medicine for the older person clinics, the day hospital service and falls and syncope clinics.

This was a convenience sample, with older people attending ambulatory services approached between October 2020 and December 2020.

### Inclusion/exclusion criteria

Participants were included if they were aged ≥70 years, had not been diagnosed with COVID-19 or admitted to hospital since March 2020 and were able to give informed consent to complete the questionnaire. The questionnaire was administered by healthcare professionals seeing the participant in the respective ambulatory care setting.

### Questionnaire

As well as collecting basic demographic information, the questionnaire covered four broad themes:

- Access to essential services, particularly healthcare services, while cocooning during the COVID-19 pandemic.
- Trend in mental health while cocooning during the COVID-19 pandemic.
- Trend in physical health while cocooning during the COVID-19 pandemic.
- Compliance with and attitude to advice regarding cocooning during the COVID-19 pandemic.

The Clinical Frailty Scale (CFS, version 2.0) was also completed on all participants.^17^ See Supplementary Material for a copy of the questionnaire.

### Statistical analysis

Data were analysed using Stata version 14.1 (StataCorp. Stata Statistical Software: Release 14. College Station, TX: StataCorp LP; 2015).

Survey data was presented descriptively as means with 95% confidence intervals and percentages. Chi-square test was used to test for differences between categorical variables.

### Ethics

The study was approved by the Tallaght University Hospital and St James’s Hospital Research Ethics Committee (Reference 2020-10).

## Results

### Baseline characteristics

Almost 55% (82/150) of participants were female, and the mean age was 79.8 years (95% CI 78.8–80.8).

Over half (78/150) of participants were married; 39% (59/150) were widowed; 8% (12/150) were single and 1% (1/150) were cohabiting. Almost half (72/150) of participants lived with a spouse or partner; 38% (57/150) lived alone while 14% (21/150) lived with family other than a spouse or partner or a friend/colleague.

The mean CFS was 4.8 (95% CI 4.6–5.0). Thirteen percent of participants (19/150) had a CFS ≤3 indicating that they were fit or managing well; 37% (55/150) had a CFS = 4 indicating very mild frailty; one fifth had a CFS =5 (28/150) indicating mild frailty and a CFS =6 (30/150) indicating moderate frailty, respectively, while the remaining 12% (18/150) had a CFS ≥7 indicating severe or very severe frailty.

### Access to healthcare

Over 57% (86/150) of participants had a scheduled healthcare-related visit cancelled while cocooning. The most frequently cancelled service was hospital outpatient appointments, with one third (50/150) of participants reporting a cancelled appointment.

Almost 1 in 6 participants reported that while cocooning they did not seek medical attention for an illness, when they otherwise would have done so. Half of those who did not seek medical attention (12/150) said this was because they were afraid of catching COVID-19 while 46% (11/150) cited a lack of the service through which they would usually access healthcare as the reason for not seeking medical attention when unwell.

### Mental health

**Figure 1.**
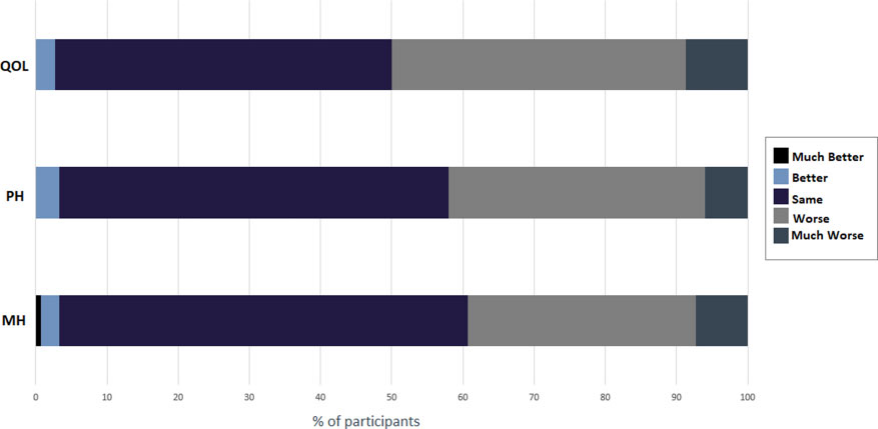
Self-reported trends in QOL, physical and mental health while cocooning. *Notes*: *n* = 150. Abbreviations: QOL, quality of life; PH, physical health and MH, mental health. Participants were asked: (1) How would you say your QOL has changed while you were cocooning? Is it Much better, better, Same/No Change, Worse or Much Worse? (2) In general, compared to before the pandemic, how would you say your physical health was while cocooning? Is it Much better, Better, Same/No Change, Worse or Much Worse? (3) In general, compared to before the pandemic, how would you say your mental heal was while cocooning? Is it Much better, Better, Same/No Change, Worse or Much Worse?

Almost 40% (59/150) of participants reported that their mental health was worse or much worse since the start of cocooning, while 57% (86/150) reported no change in their mental health and 3% (5/150) reported an improvement in their mental health since they were advised to cocoon (Figure 1).

**Figure 2.**
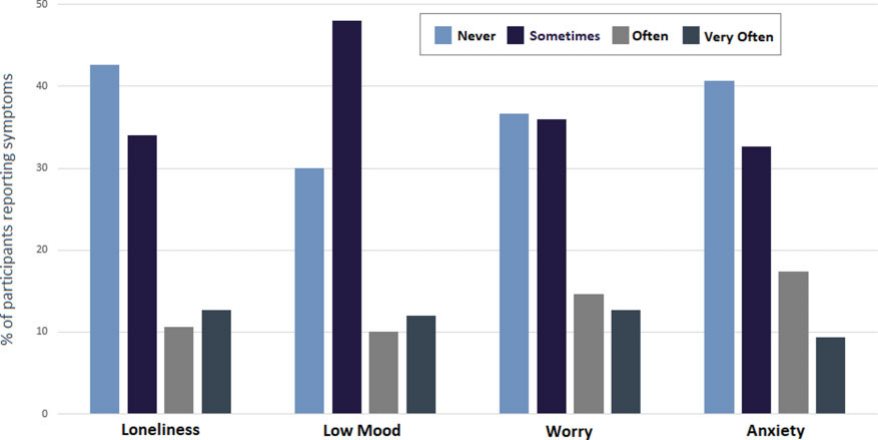
Reported prevalence of loneliness, low mood, worry and anxiety while cocooning. *Notes*: *n* = 150. Participants were asked: How would you say your mobility/fitness/energy levels/sleep/diet has changed while you were cocooning? Is it Much Better, Better, Same/No Change, Worse or Much Worse?

Figure 2 demonstrates the reported prevalence of loneliness, low mood, worry and anxiety amongst participants during cocooning.

Over 57% (86/150) of participants reported loneliness at least some of the time while cocooning with 1 in 8 (19/150) participants reporting that they were lonely ‘very often’. Seventy percent (105/150) of participants reported low mood at least some of the time, with 12% (18/150) reporting low mood ‘very often’.

Participants were almost twice as likely to report loneliness if they lived alone (47% vs. 27%; *X*^2^ = 6.20; *P* = 0.013).

### Physical health

Over 40% (63/150) of participants reported a decline in their physical health since cocooning, while 55% (82/150) reported no change in their physical health and 3% (5/150) reported an improvement in their physical health status (Figure 1).

Of those reporting a decline in physical health, one third (21/63) reported not leaving the house at all while cocooning, compared to 10% (9/87) of those who did not report a decline in physical health (*X*^2^ = 12.07; *P* = 0.001).

**Figure 3.**
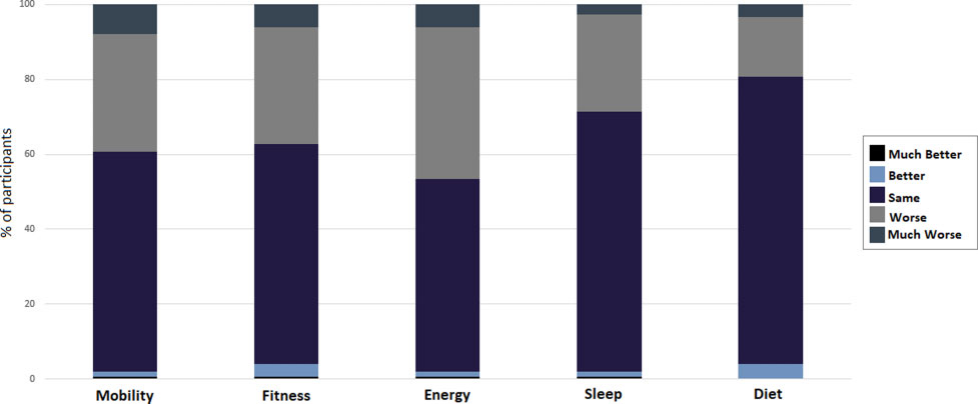
Self-reported change in physical health parameters while cocooning. *Notes*: *n* = 150. Participants were asked: How would you say your mobility/fitness/energy levels/sleep/diet has changed while you were cocooning? Is it Much Better, Better, Same/No Change, Worse or Much Worse?

Figure 3 demonstrates the changes in physical health parameters reported by participants while cocooning.

Almost 40% (59/150) reported a decline in their mobility since cocooning, with 8% (12/150) reporting their mobility was ‘much worse’. Over one third (56/150) felt their physical fitness had declined and almost half (70/150) of participants reported lower energy levels since beginning cocooning. Almost one third (43/150) reported a decline in the quality of their sleep and one fifth (29/150) reported a worse diet.

### Quality of life

Half of participants (75/150) reported a decline in their quality of life (QOL) while cocooning. Three percent (4/150) reported an improved QOL, while the remaining participants (71/150) noted no change in their QOL. Almost 10% (13/150) reported that their QOL was ‘much worse’ than prior to the pandemic (Figure 1).

QOL was more likely to decline in those who also reported a decline in mental health (*X*^2^ = 17.46; *P* < 0.001) and physical health (*X*^2^ = 33.52, *P* < 0.001) or who reported loneliness (*X*^2^ = 10.90; *P* = 0.001). There was no association between poorer QOL and living alone (*X*^2^ = 1.39; *P* = 0.239), family visiting less frequently (*X*^2^ = 1.32; *P* = 0.251) or QOL and not leaving the house at all while cocooning (*X*^2^ = 0.67; *P* = 0.414).

### Attitudes to and compliance with COVID-19 restrictions

Over half (81/150) of participants reported seeing their family members less frequently since being advised to cocoon. Three percent (5/150) reported seeing their families more during this time.

One in five (30/150) reported not leaving their house at all since being advised to cocoon, while over 60% (92/150) left the house less often and a further one in five (28/150) reported leaving the house as frequently as before.

Over half (79/150) of participants reported not seeing friends or colleagues at all since being advised to cocoon, while a further 38% (57/150) saw friends less frequently. Less than 1 in 10 reported seeing friends as frequently as before.

One-quarter of participants (41/150) reported not exercising at all, 42% (63/150) reported exercising less frequently than before, 29% (43/150) reported exercising the same amount of time, while 2% (3/150) reported exercising more frequently while cocooning.

Over 60% (91/150) reported not using public transports at all, while one third (51/150) reported not doing grocery shopping at all since being advised to cocoon.

One quarter (39/150) of participants reported that they did not agree with the government advice regarding cocooning. Almost 17% (25/150) strongly agreed with cocooning, 44% (66/150) agreed and 13% (20/150) reported that they neither agreed nor disagreed with the government advice.

There was no association between reported loneliness (*X*^2^ = 1.99; *P* = 0.158), decline in mental health (*X*^2^ = 0.07; *P* = 0.786) or decline in physical health (*X*^2^ = 0.01; *P* = 0.941) with the level of agreement with advice to cocoon. There was also no association between the frequency of times participants left the house while cocooning and reported agreement with cocooning advice (*X*^2^ = 0.25; *P* = 0.616).

Over 40% (61/150) of participants reported that they disliked the term ‘cocooning’ however, while almost 10% (14/150) reported that they liked the term.

Over half (77/150) of participants were not in favor of ‘virtual’ clinics over the telephone or via video call, while one quarter (38/150) were in favor of such clinics.

## Discussion

The study involved a convenience sample of older adults attending specialist ambulatory medical services who were not acutely unwell and examined changes in their physical and mental health while cocooning during the COVID-19 pandemic. We also explored the effect the pandemic has had on their access to healthcare, as well as the compliance to and attitudes toward COVID-19 restrictions amongst this cohort.

Self-reported mental health declined significantly while cocooning. We found that 2 in 5 participants reported a decline in their mental health overall, with 70% reporting low mood at least some of the time and 12% reporting low mood very often. Three in five participants reported loneliness and loneliness was twice as prevalent in those living alone than those living with spouses or other family members.

These findings are consistent with other studies demonstrating a decline in mental health amongst older people during the COVID-19 pandemic. In the UK Household Longitudinal Study, the prevalence of clinically significant mental distress rose from 11% to 18% from 2018–19, prior to the COVID-19 pandemic, to April 2020 amongst participants aged ≥70 years.^18^ In Ireland, perceived stress amongst adults aged ≥60 years increased by 20% post-pandemic.^19^ Pre-existing health conditions, high estimates of personal risk and time spent quarantining, all of which are more prevalent in those aged ≥ 70 years, appear to be independent risk factors for depression during the COVID-19 pandemic.^20^^,^^21^

Additionally, over 40% of participants reported a decline in their physical health while cocooning. Participants reporting a decline in physical health were three times more likely to also report not leaving the house at all since being advised to cocoon. Almost 40% of participants noted a decline in their mobility and 40% reported having lower levels of fitness while cocooning. Almost half of those surveyed reported lower energy levels and over one quarter reported poorer sleep while cocooning. Half of participants reported a decline in QOL.

Given the constraints imposed by cocooning on social interaction and physical activity, these findings are not necessarily surprising. Maintenance of social engagement is strongly associated with better self-reported health status and lower burden of depressive symptoms in later life^22^ while physical activity is associated with better mental health,^23^ QOL^24^ and better health trajectories in general.^25^ A period of 3 months detraining in older adults who previously exercised regularly is associated with a significant decline in standing balance, gait and QOL for example.^26^

Despite these poorer health trajectories while cocooning, over 60% of participants agreed with government advice regarding cocooning and there was a high degree of reported compliance with this advice, even amongst those who also reported declining physical or mental health. Worryingly, one in six participants also reported that they avoided seeking medical attention when unwell while cocooning, with concern regarding contracting COVID-19 or inability to access appropriate clinical pathways cited as common reasons.

There are some limitations to this study that should be noted. While participants were asked about health trajectories it is beyond the scope of the study to determine if decline in health status was directly related to cocooning or for other reasons but participants who were diagnosed with COVID-19 or those who were admitted to hospital during the COVID-19 pandemic were excluded. All participants were surveyed while in a healthcare setting, and responses regarding compliance with government advice and attitudes to COVID-19 restrictions must be interpreted in this context, as for example, it is possible that some may have been reluctant to report non-compliance in this context. Further, all measures of health are by self-report only and further studies with objective health measures would therefore be welcome. The strengths of this study include the fact that it involves a sample of older adults attending ambulatory medical services and therefore with relatively high rates of frailty and comorbid disease. Surveys were completed face-to-face, rather than online or virtually and to the authors knowledge, this is the first study conducted on a clinical sample of older adults who did not contract COVID-19 regarding health trajectories while cocooning or shielding.

These findings highlight the potential secondary impact of the COVID-19 pandemic on older people. While cocooning or shielding reduces the likelihood of older people contracting COVID-19, there may be important adverse impacts on the health of those who cocoon that need to be addressed. Given the likelihood of further waves of COVID-19 in coming months, with the possibility of further restrictions despite the rollout of vaccines, clear policies and advice for older people around strategies to maintain social engagement, manage loneliness, continue physical activity and avoid deferring the need for medical attention when unwell should be a priority, as well as a focus on provision of appropriate rehabilitation services for this cohort.

## Supplementary material

Supplementary material is available at *QJMED* online.

## Supplementary Material

hcab015_Supplementary_Data
