# Supplementary material for: Physical and mental health of older people while cocooning during the COVID-19 pandemic
Source: QJM. 2021 Jan 20;114(9):648–53. doi: 10.1093/qjmed/hcab015 (PMC7928635; doi:10.1093/qjmed/hcab015)
Supplement: hcab015_Supplementary_Data [file hcab015_supplementary_data.docx]

**Patient Survey on ‘Cocooning’ during the Covid-19 Pandemic**

*[The following questions refer to the period of ‘cocooning’, which is the term commonly used to describe how people aged 70 years or over were advised to stay at home and reduce face-to-face interaction with other people as much as possible, in order to reduce the risk of contracting Covid-19. This began in late March 2020 and these recommendations, with removal of some restrictions, remain in place now. We want to see how your health changed, for better or worse, while cocooning]*

Diagnosed with Covid-19: Yes No

CFS: 1 2 3 4 5 6 7 8 9

Are you: Married Widowed Single Cohabiting

Living: Alone With Spouse / Partner With Family

Prior to cocooning how often would you have seen your family (from outside your household/home)?

No immediate family Everyday >2 per week Weekly Monthly Less often

During cocooning how often did you see your family (from outside your household/home)?

No immediate family Everyday >2 per week Weekly Monthly Less often

**A) Healthcare Use**

Since cocooning began in March 2020, have you had any of the following cancelled/deferred: [*CIRCLE ANSWER]*

1. Hospital outpatient appointment

2. Hospital procedure such as an operation or camera test

3. Home Help

4. Visits for personal care from care agency/ careworker

5. Other appointments such as:

a. wound dressing

b. blood tests, including warfarin

c. general practitioner review

d. public health nurse review

e. chiropody

f. meals on wheels

g. day centre services

h. physiotherapy/occupational therapy/speech therapy

i. psychological/counselling services

j. respite

k. other service

At any stage while cocooning have you avoided seeking medical attention when you would otherwise have done so? Yes No

If yes, why?

Service cancelled afraid of catching COVID no carer to help/drive to appointment.

**B) Mental Health**

How often have you experienced the following while cocooning?

*[CIRCLE ANSWER]*

1. Loneliness Never Sometimes Often Very Often

2. Low mood Never Sometimes Often Very Often

3. Anxiety Never Sometimes Often Very Often

4. Worry Never Sometimes Often Very Often

5. Poor Sleep Never Sometimes Often Very Often

6. In general, compared to before the pandemic, how would you say your mental health was while cocooning?

Much Better Better Same/No Change Worse Much Worse

**C) Physical Health**

How would you say any of the following changed while you were cocooning?

*[CIRCLE ANSWER]*

1. Mobility Much Better Better No Change Worse Much Worse

2. Physical fitness Much better Better No change Worse Much Worse

3. Quality of Life Much Better Better No Change Worse Much Worse

4. Energy Levels Much Better Better No Change Worse Much Worse

5. Sleep Much Better Better No Change Worse Much Worse

6. Diet Much Better Better No Change Worse Much Worse

7. Ability to Dress Much Better Better No Change Worse Much Worse

8. In general, compared to before the pandemic, how would you say your physical health was while cocooning?

Much Better Better Same/No Change Worse Much Worse

**D) Other Services/Activities**

Since cocooning, how often do you do the following activities, as compared to beforehand?

*[CIRCLE ANSWER]*

1. Leave your home Not at all Less Often About The Same More Often

2. Meet friends Not at all Less Often About The Same More Often

3. Grocery shopping Not at all Less Often About The Same More Often

4. Attend religious services Not at all Less Often About The Same More Often

5. Exercise Not at all Less Often About The Same More Often

6. Attend a social club/group Not at all Less Often About The Same More Often

6. Go to the pub Not at all Less Often About The Same More Often

7. Go to a restaurant Not at all Less Often About The Same More Often

8. Use public transport Not at all Less Often About The Same More Often

**E) Attitudes to Covid-19/Restrictions** *[CIRCLE ANSWER]*

Do you agree with the government’s advice re cocooning for those over 70 years?

Strongly agree

Agree

Neither agree nor disagree

Disagree

Strongly disagree

With the introduction of Covid-19 restrictions healthcare visits, such as general practitioner appointments and hospital clinics are now frequently being done via phone or video call, so called virtual clinics. How do you feel about that?

I am not in favor of virtual clinics

I am in favor of virtual clinics

I am neither in favor nor not in favor of virtual clinics

How do you fell about the term ‘cocooning’?

I like it

I dislike it

I neither like nor dislike it
